# Spinning and corkscrewing of oceanic macroplankton revealed through in situ imaging

**DOI:** 10.1126/sciadv.adm9511

**Published:** 2024-05-15

**Authors:** Kelly R. Sutherland, Alejandro Damian-Serrano, Kevin T. Du Clos, Brad J. Gemmell, Sean P. Colin, John H. Costello

**Affiliations:** ^1^ Oregon Institute of Marine Biology, University of Oregon, Eugene, OR 97405, USA.; ^2^ Louisiana Universities Marine Consortium, Chauvin, LA 70344, USA.; ^3^ Department of Integrative Biology, University of South Florida, Tampa, FL 33620, USA.; ^4^ Marine Biology/Environmental Sciences, Roger Williams University, Bristol, RI 02809, USA.; ^5^ Whitman Center, Marine Biological Laboratory, Woods Hole, MA 02543, USA.; ^6^ Biology Department, Providence College, Providence, RI 02908, USA.

## Abstract

Helical motion is prevalent in nature and has been shown to confer stability and efficiency in microorganisms. However, the mechanics of helical locomotion in larger organisms (>1 centimeter) remain unknown. In the open ocean, we observed the chain forming salp, *Iasis cylindrica*, swimming in helices. Three-dimensional imaging showed that helicity derives from torque production by zooids oriented at an oblique orientation relative to the chain axis. Colonies can spin both clockwise and counterclockwise and longer chains (>10 zooids) transition from spinning around a linear axis to a helical swimming path. Propulsive jets are non-interacting and directed at a small angle relative to the axis of motion, thus maximizing thrust while minimizing destructive interactions. Our integrated approach reveals the biomechanical advantages of distributed propulsion and macroscale helical movement.

## Teaser

Open ocean observations reveal colonies of salps swimming in giant helices through coordinated jet propulsion.

## INTRODUCTION

Animals have evolved a diversity of movement patterns for locomotion that underpin ecological strategies. Some of the most prevalent long-distance vertical migrators in the open ocean are colonial swimmers composed of multiple individuals who cooperate as an integrated whole. Salps, a type of pelagic tunicate, are one such colonial swimmer. Salps use multiple pulsed swimming jets, produced by individual zooids that are neurologically integrated (*1*) and conjoined in a colony often shaped as a chain (*2*). In the open ocean, we observed salp colonies of the species *Iasis cylindrica* swimming in helices. Despite their prevalence, we lack a cohesive framework to explain how these teams of individuals achieve joint success.

For microorganisms with cilia and flagella operating at low Reynolds numbers (*Re*), helical swimming, where organisms swim in a corkscrew pattern, is a common mode of locomotion (*3*). Detailed studies of these microswimmers (swimming microorganisms) provide a useful analog for helical swimming at higher *Re*, establishing the extent to which spiral motions create stable and efficient locomotion dynamics. Helical swimming in protozoans and ciliated larvae has been recognized for over a century (*4*) and continues to be an active area of research (*5*, *6*). The morphologies, swimming kinematics, and phylogenetic origins of these helical microswimmers are diverse, as they include bacteria (*7*), eukaryotic algae (*8*, *9*), sperm (*10*), parasitic flagellates (*11*), ciliates (*12*), and multicellular larvae (*13*). For example, the unicellular alga, *Chlamydomonas*, swims in a helical pattern by occasional asynchronous beating of its two flagella (*9*). Ciliates swim in a right-handed helix due to ciliary beat patterns (*14*). These periodic, asymmetric beat patterns, termed the helix theorem (*15*), help microswimmers orient to chemical gradients (*16*). This unique mode of movement has inspired robotic helical microdevices that can navigate complex environments with diverse applications (*17*, *18*).

**Fig. 1.**
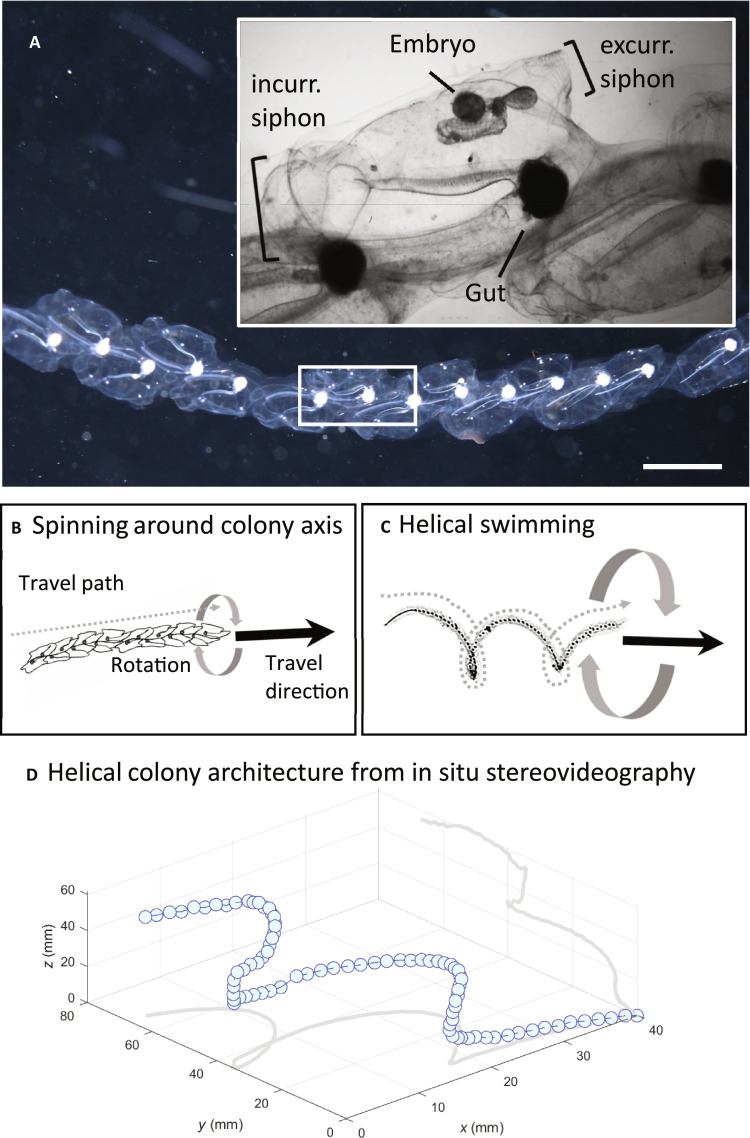
Morphology and swimming postures of the salp *Iasis cylindrica*. Overall colony structure (dark-field image) with inset (bright-field image) showing individual zooid morphology (**A**); sketches showing spinning, where the colony rotates around the central chain axis (**B**); and helical swimming (**C**), where the colony swims in a helical path. Black arrows show the net swim direction, dotted lines show the swimming path, and curved arrows show the rotation of the colony. (**D**) 3D colony architecture derived from in situ stereo videography with projections shown in *x*-*y* and *y*-*z* planes. Scale bar, 1 cm (A).

An advantage of these microorganisms as propulsive models is that their size enables precise microscopic measurements in the laboratory. However, these models may not account for the whole range of sizes and flow dynamics in which helical swimming can occur in nature. During hundreds of hours collectively spent underwater, we have observed spiral swimming patterns in many colonial animal migrators in the open ocean. These organisms are substantially larger (centimeter-to-meter length) and use very different thrust production—typically jet propulsion—compared to the flagella and cilia of microswimmers. Nonetheless, the results are similar—successful forward motion—but the underlying mechanics for spiraling by these larger swimmers are unknown. One of the most notable examples of helical swimming we have observed is found in salp colonies that are centimeters long (10 to 30 cm) (Fig. 1).

Individual salp zooids are barrel-shaped and swim using pulsed jets where muscular contractions pull fluid in through an anterior incurrent siphon and then expel fluid through the rear, exhalant siphon (*2*). Pulsatile jet propulsion is a highly effective locomotory mechanism among animals (*19*, *20*), but salps are unique in having openings at opposite ends, facilitating forward and reverse locomotion. The colonial stage zooids of many salps species, such as *Salpa*, *Iasis*, *Soestia*, *Metcalfina*, and *Ihlea* spp. (*21*), are connected to form streamlined linear chains, and, as chains, they experience further benefits of drag reduction and steady swimming (*22*). In these linear chains, asynchronous pulsing by the individual zooids preserves the advantages of swimming by jet propulsion while simultaneously reducing the increased drag associated with periodic acceleration and deceleration. Swimming in a helix represents a higher-order, colony-level swimming feature that may further enhance or reduce swimming performance. Determination of the mechanics that generate successful spinning propulsion by individuals within these animal colonies is required to understand how colonies perform their long-distance movements. This knowledge may also contribute to the design of multi-propulsor devices that must traverse similar distances and conditions as these animals.

Although widespread in the world’s oceans, salps are notoriously more difficult to study than model microswimmers. Most species of salps are restricted to the open ocean and therefore challenging to access and study (*23*). Moreover, these animals are challenging to keep alive in captivity (*24*). Hence, even when chains are sampled painstakingly to keep them intact, their extreme fragility requires observations in large volumes of water soon after collection. Consequently, empirical measurements of salp colony morphology and swimming have been elusive.

We translated three-dimensional (3D) optical techniques developed in the laboratory to in situ conditions where the animals behave naturally. We used bluewater SCUBA dives in the open ocean and diver-collected specimens to reveal the form and function of a representative salp species (*I. cylindrica*) with linear colony architecture and helical swimming behavior.

## RESULTS

We imaged a total of 66 chains in situ and 31 hand-collected chains in the lab (means ± SD: zooid number = 39 ± 36, *n* = 75; chain length = 153 ± 94 mm, *n* = 40, zooid length = 10 ± 5 mm, *n* = 43) working from three oligotrophic, open-ocean field sites: West Palm Beach, FL, USA (26°43′93″N, 79°59′15″W; July, 2021), Kailua-Kona, HI, USA (19°42′38.7″N 156°06′15.8″W; April 2022), and the Pacific coast of Panama, Veraguas province (7°50′N, 81°35′W; February, 2017). We used high-resolution, 3D imaging to describe the colony morphology, movement, and fluid mechanics that underpin helical swimming (data S1).

### Helical swimming derives from the oblique orientation of zooids

**Fig. 2.**
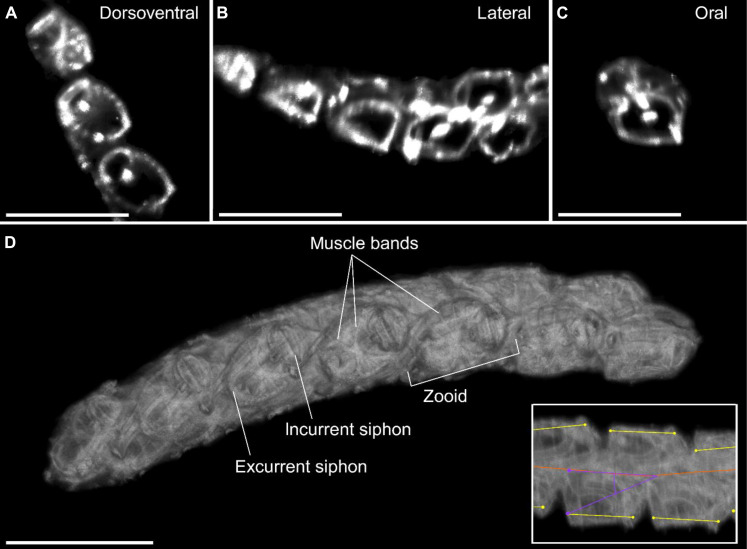
Three-dimensional colony architecture of *Iasis cylindrica*. Laser scans show colony arrangements from dorsoventral, lateral, and oral planes (**A** to **C**). 3D reconstruction from scans shown here from the dorsoventral view yields additional detail, including offset in the dorsoventral and lateral planes (**D**) as highlighted in the inset of the zooid from a lateral view. The inset indicates the locations of the midline of the chain (red), the dorsoventral zooid angle (yellow), and the lateral angle of the excurrent siphon with respect to the chain axis (purple). Scale bars, 1 cm. See also movie S1.

Using a high-resolution laser scanning system, we recorded three to five scans each of four freshly collected *I. cylindrica* colonies (a total of 19 scans). Here, we present a representative scan revealing the 3D architecture of the chains, as well as the external and internal morphology of individual zooids (Fig. 2 and movie S1). The reconstructed scan showed that zooids of *I. cylindrica* are interlocked with their serial neighbors and are obliquely oriented relative to the swimming axis of the chain in the dorsoventral plane. Spinning and swimming in helices can arise from asymmetry in body form or kinematics (*15*). Our 3D reconstructions indicate that the intrinsic shape of the zooids and the oblique chain architecture in the dorsoventral plane angle the jets in a way that generates an asymmetry of forces during swimming. Since the zooids present only chiral (not rotational) symmetry with their ventral neighbors, this asymmetry is not offset. The off-axis force created by the angle of the zooids relative to the chain axis provides the torque that initiates a spinning motion. This torque likely creates enough instabilities to ultimately cause buckling into a helical form as chains increase in length.

### Chain architecture gives rise to spinning and corkscrewing

**Fig. 3.**
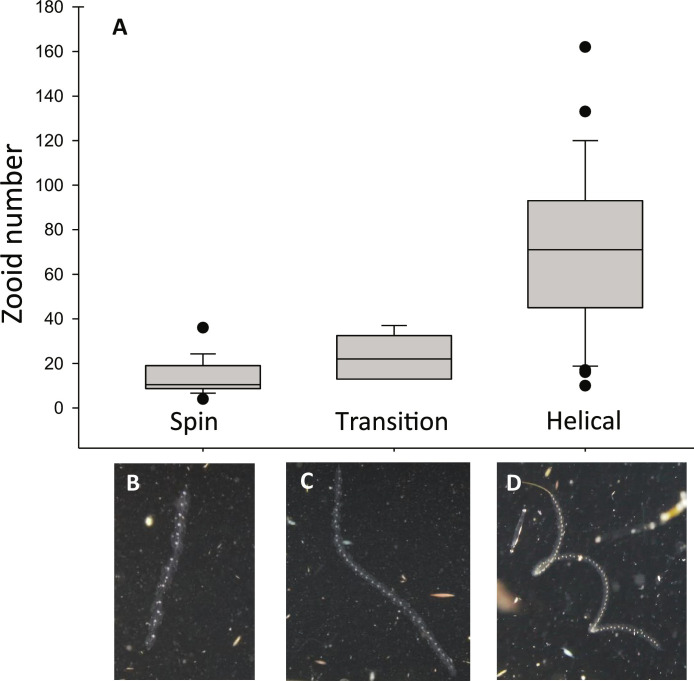
Colony size drives the transition from spinning to helical swimming. Zooid number versus swimming mode (**A**). Shorter chains with fewer zooids swim straight and spin around a central axis (**B**), while longer chains transition (**C**) to swimming in a helix (**D**). The number of zooids per colony differed significantly across the three swimming modes: spinning (means ± SD = 13.8 ± 7.7, *n* = 18), transition to helical (23 ± 9.9, *n* = 6), and helical swimming (70.0 ± 36.3, *n* = 31) (ANOVA, *P* < 0.001). In situ images show the posture of the colony during different swimming modes (see also movies S2 and S3).

**Fig. 4.**
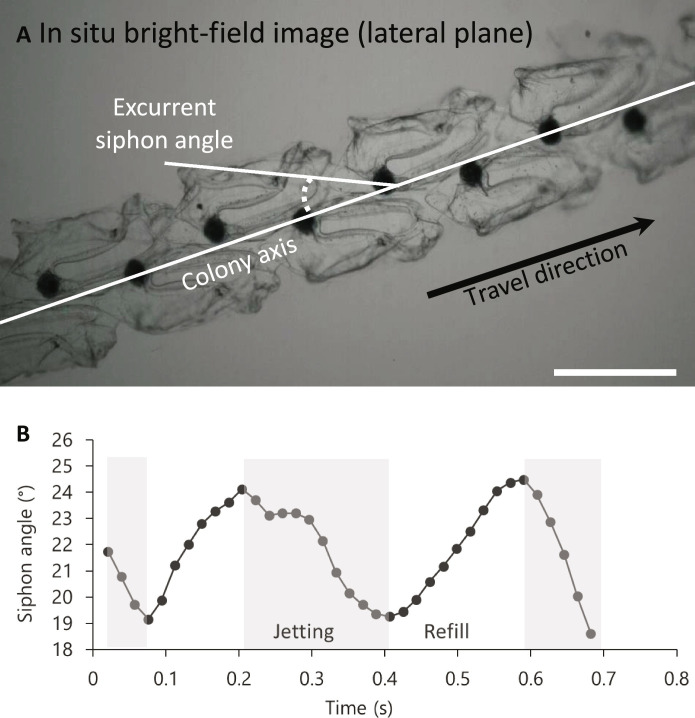
Colony architecture dictates movement. Two-dimensional lateral view (in situ bright-field image) shows low siphon lateral angles relative to the chain axis (means ± SD = 20 ± 2°, *n* = 8) (**A**); siphon angles are time-varying with siphons forming the lowest angles relative to the chain axis during jetting (**B**). Scale bar, 1 cm. See also movie S4.

Imagery from a custom-built stereo camera system showed colony shape (Fig. 1D) together with swimming paths, swim speeds, and spinning orientations of chains in three dimensions. Colonies translated through the water at a speed of 178.8 ± 205.3 mm s^−1^ (*n* = 18). Shorter chains (means ± SD: zooid number = 14 ± 8, Fig. 3) spin (rotate) around the colony axis of the chain (Fig. 4A), which in this case lies centered and parallel to the axis of locomotion (movies S2 and S3). Short chains swam in a linear path and rotated at a rate of 162 ± 68.5 radians s^−1^ (*n* = 13) but the direction of spinning was variable with both clockwise (33%, *n* = 5) and counterclockwise (66%, *n* = 10) spinning observed. We did not observe any chains spinning in both directions. The variability in spinning direction suggests that, in addition to chain architecture, spinning can be mediated through zooid control of the jet by excurrent siphon kinematics. We found that the direction of spinning could not be discerned from a single two-dimensional image sequence due to biased rotation perception termed “the spinning dancer illusion” (*25*). In other words, two observers viewing the same video could perceive different spinning directions. Therefore, image analysis of stereo pairs was crucial to accurately determine the direction of spinning. It has proven challenging to discern the rotational direction of helical-swimming microorganisms because they move in an out of the focal plane. Therefore, controversy remains about whether the direction is left-handed or right-handed or switches periodically (*16*). Macroscopic salps present a more tractable case for measuring the rotational direction of spinning via stereovideography.

Longer chains (means ± SD: zooid number = 70 ± 36; Fig. 3) swam in a helical swimming path orbiting around a linear axis of displacement, a mode of swimming that has been described extensively in microorganisms but has not been documented in larger organisms (>1 cm; movies S2 and S3). Although the chain assumed a corkscrew (helix) shape and each zooid traced a helical path, the path of the colony displacement at a larger scale was linear, i.e., the axis of the helix was straight (Fig. 1C). Intermediate length chains (means ± SD: zooid number = 23 ± 10) were transitional between spinning and corkscrew, dividing their time between spinning and swimming in a helix or partial helix.

### Orientation of propulsive jets maximizes thrust

Although zooids have an intrinsic shape and relative orientation that together determine the architecture of the colony, the effective shape taken by the flexible chain as it swims is determined by its movement. The dorsoventral angle of the zooid’s oral-aboral axis relative to the elongation axis of the chain is not perfectly parallel; instead, zooids are tilted at an oblique angle (means ± SD: 22.3° ± 5.3°; range: 12.8° to 29.6°; *n* = 12), a feature that is only fully apparent from 3D imaging (Fig. 2 and movie S1). From the lateral view (Fig. 4A), bright-field imagery showed that zooids are also tilted at an angle relative to the colony axis (means ± SD: 22.2° ± 6.0°; range: 16.3° to 37.3°; *n* = 9). The offset in both the lateral and dorsoventral angle relative to the chain axis means that swimming jets emerging from the excurrent siphons are less likely to be inhaled by the incurrent siphon of the serial neighbor zooid downstream. This is advantageous for filter-feeding efficiency since the same fluid that creates jet wakes also contains planktonic food that is filtered by the zooid using a mucus mesh that fills most of the barrel-shaped body (*23*). In salps a slight offset (i.e., an absolute angle > 0°) in the dorsoventral plane helps ensure that the effluent of a zooid is not taken up by its neighbors without requiring larger jet angles in the lateral plane that would reduce the thrust-to-torque ratio. The zooids in the present study had reached their final adult orientation but zooids from newly released colonies have higher dorsoventral angles—near 60°—relative to the chain axis when they are small (~3 to 4 mm) and become more linear through development (*26*). Therefore, these smaller zooids experience more torque.

**Fig. 5.**
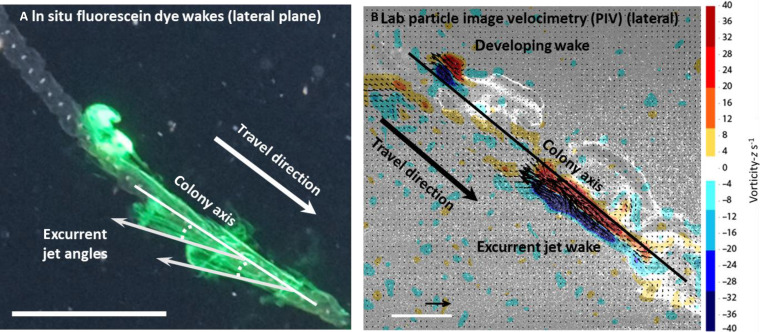
Low lateral jet angles maximize thrust. In situ jet wakes visualized using fluorescein dye show that jets emerge at a low jet angle in the lateral view (**A**) and lab particle image velocimetry (**B**) corroborates that jets are almost parallel to the chain axis and reach velocities of 15 cm s^−1^. Scale bars, 10 cm (A) and 1 cm (B). Vector scale, 10 cm s^−1^. See also movies S5 and S6.

The time-varying lateral zooid-colony angle measured from a single colony using in situ bright-field videography decreased by 21.2% from a maximum of 24.5° to a minimum of 19.3° relative to the chain axis during the jetting period of the zooid’s pulsatile cycle (Fig. 4 and movie S4). Low angle jets maximize forward thrust and minimize torque, or turning (*27*, *28*). The positions of the zooids and time-varying lateral excurrent siphon angles were corroborated with measurements of the jet wakes using fluorescein dye in situ and particle image velocimetry (PIV) in the laboratory (Fig. 5 and movies S5 and S6). Swimming jets produced by pulsing zooids were directed at a low lateral angle (means ± SD: 21.4° ± 6.1°; range: 16.3° to 29.9°; *n* = 6) relative to colony axis from the lateral view of the zooids. Jet wake lateral angles were similar to the minimum fixed angle of the excurrent siphon (Figs. 4 and 5), suggesting that siphon movement plays an important role in directing jets to be closer to parallel to the colony axis in the lateral plane. The jet wake angles are even lower than those of the siphonophore *Nanomia bijuga*, another colonial multijet swimmer, that produces jets that are at a minimum of 40° relative to the colony axis (*27*). *N. bijuga* has a similarly elongated colony morphology and may also spin during swimming. Thus, the higher jet angles in *N. bijuga* may serve to stabilize the colony while simultaneously providing forward thrust. In siphonophores, the oblique angle of the jets may also help shield the feeding zooids, which are situated below the swimming zooids, from hydrodynamic disturbance (*28*). Moreover, *N. bijuga* nectophores can direct the angle of these jets to maximize torque during sideways maneuvers and turning.

Salp jets emanating from the narrow exhalent siphon with diameter, *L* = 0.3 mm reached speeds of *U =* 15 cm s^−1^ (Fig. 5 and fig. S1) corresponding to a dimensionless Reynolds number (*Re* = *Ud/v*) of 429, where kinematic viscosity, *v* = 1.05 × 10^−6^ m^2^ s^−1^. The ejected fluid rolled up into a vortex ring with a minimal trailing jet, consistent with the concept of optimal formation number (*F*) (*29*). Despite their close spacing, previous dye observations showed that salp jet wakes did not interact (*22*). A lack of interaction between jets has been described as resulting from jets emerging out of the time phase during asynchronous swimming, but the chain morphology behind non-interacting jet wakes was not understood. Closely spaced, in-phase (synchronous) jets produced in the laboratory resulted in a reduction in thrust (*30*), highlighting the advantage of non-interacting jet wakes for enhanced swimming performance. The oblique zooid orientation together with pulse timing ensures that jet wakes do not interact in space or time even when all zooids pulse synchronously during an escape response.

Disentangling the relative contribution of morphology and kinematics toward helical swimming performance in salp chains will require further investigation. In siphonophores, speed and efficiency increase as more swimming units are added. In addition, the coordination of the zooids produces two swimming modes: synchronous swimming yields higher speeds and accelerations, while asynchronous swimming is more efficient (*31*). Future observations, modeling studies, or experiments with robotic salps will enable explicit hypothesis testing and evaluation of performance across parameter space, including zooid number and size, synchronous versus asynchronous coordination of pulses, forward versus backward swimming, and spinning versus helical motion.

## DISCUSSION

### Helical swimming may be pervasive in the open ocean

spinning and helical swimming are not constrained to *I. cylindrica.* During hours of in situ oceanic work, we have observed other species of salps (*Salpa* spp., *Soestia zonaria*, and *Riteriella* spp.) as well as organisms from distinct evolutionary lineages, spinning and coiling (e.g., alciopid worms, cnidarian siphonophores, and the ctenophore *Eurhamphaea vexilligera*). Yet, descriptions of helical swimming have been constrained to more tractable microorganisms (<1 mm). Colonial animals such as salps and siphonophores are comprised of fragile gelatinous bodies that are not readily maintained in laboratory conditions. To study them, we translated laboratory optical methods to in situ conditions to capture natural behaviors. We present a detailed description of helical swimming in a large colony (>1 cm) with multiple, identical swimming units. Our methodologically nested approach revealed the morphology of the zooids, their arrangement into chains, the movement of the colonies, and the resultant jet wake structures underlying their helical swimming.

The discovery of spinning and helical swimming in salps described here represents a unique mode of locomotion in the ocean that has not been described for organisms operating at higher *Re*. Spinning in the open ocean may be more pervasive than currently appreciated as in situ imaging techniques reveal the true abundance of fragile gelatinous forms in the open ocean and midwater [e.g., (*32*)], ecosystems that comprise most of Earth’s habitable volume (*33*). Spinning around an axis stabilizes locomotion and therefore produces straight swimming paths. For certain behaviors, such as swimming up a gradient or long-distance vertical migrations, swimming in a straight path minimizes the metabolic demands of travel. Considering that directed swimming may be critical for survival, swimming performance can constrain the evolution of zooid morphologies and chain architectures (*21*). The in situ imaging approaches used here can be applied to other elusive free-swimming animals in the ocean to reveal complex details of their morphology, movement, and fluid mechanics.

## MATERIALS AND METHODS

### Bluewater diving at field sites

*I. cylindrica* colonies were filmed or collected by hand in 1-liter jars via bluewater diving from small dive vessels (*34*). Dives were conducted in the top 25 m and between 6 and 8 km from shore in oceanic, oligotrophic waters in (i) the Atlantic Gulf Stream near Palm Beach, Florida, USA (26°43′93″N, 79°59′15″W; July 2021), (ii) the north Pacific subtropical gyre near Kailua-Kona, Hawaii, USA (19°42′38.7″N 156°06′15.8″W), and (iii) in the eastern tropical Pacific Ocean near Santa Catalina, Veraguas, Panama (7°50′N, 81°35′W; February 2017). As non-vertebrates, *I. cylindrica* colonies are exempt from standards of care under the Institutional Animal Care and Use Committee procedures and are not protected by state or federal law.

### Three-dimensional morphology from laser scans in the laboratory

Because of the 3D nature of colonies, 2D imaging does not fully resolve the arrangement of individual zooids. Therefore, we used a laser scanning system to image the colonies [similar to the system described in (*35*)]. Colonies in this study were carefully hand-collected individually in jars along with water from the collection location. Back in the laboratory, colonies were placed in a custom-built 20-liter acrylic tank of water from the field within 12 hours of animal collection. To prevent motion blur from swimming, we anesthetized the colonies using 0.2% MS-222. A high-speed camera (Photron Nova R2) was used to image the animal, and illumination was provided by a green laser (Hercules, 532 nm) with a sheet-forming optic to provide a 1-mm-thick laser sheet. The camera and laser sheet were mounted perpendicular to each other on a motorized linear translation system (B&H) and translated through the tank at a constant rate. Each frame of the video represents a slice of the volume along the *z* axis. To reconstruct morphology from video scans, we used the free and open-source packages Ilastik (*36*) and 3D Slicer (*37*, *38*).

### In situ stereovideography to measure whole-colony, 3D swimming patterns

Because salp colonies swim in three dimensions, we recorded their swimming patterns using an in situ dark-field stereovideography system comprising two synchronized high-resolution cameras (Z Cam E2; 4K at 160 fps) housed in custom aluminum housings (Sexton Company). The image from the right-hand camera was viewed using an external monitor (Aquatica Digital), and illumination was provided with two 10,000-lumen lights (Keldan). An L-shaped plastic framer helped the videographer position colonies in the field of view of both cameras. Before diving, the stereo system was calibrated in a swimming pool using a cube with reflective landmarks. Calibration images were processed using the CAL software package (SeaGIS measurement science).

Calibrated stereo images of swimming *I. cylindrica* chains were analyzed in EventMeasure (SeaGIS) for morphology and kinematics. Zooid length and width, zooid number, and chain length were measured from single image pairs. The *x*, *y*, and *z* coordinates of chains were tracked manually through multiple images using the gut (viscera or nucleus) of the first zooid as a landmark. To account for the background motion of the water, the diver, or the camera, we also tracked a non-motile reference particle (i.e., marine snow, radiolarian, or foraminiferan) in close proximity to the chain. Resultant swimming trajectories over time *t* and speeds *U* were calculated by subtracting the coordinates of the reference particle at each frame to calculate motion-corrected coordinates, *x_adj*, *y_adj*, and *z_adj*.

The swimming posture of the chain was coded as either spinning around a linear axis (orbit radius of 0 mm), swimming in a full helix (orbit radius larger than 0 mm), or transitioning between spinning and helical modes. For chains spinning around a linear axis, the angular velocity was calculated by first defining the linear axis of the chain by digitizing the first four zooids in the chain and then identifying a reference landmark on the outer edge of one of the zooids (usually an unreleased fecal pellet or parasitic amphipod). The motion of the off-axis reference landmark relative to the linear axis of the chain (defined by the shortest line between the frontal zooid gut and the adjacent zooid gut) was then used to calculate the direction of spinning and the angular velocity as the change in instantaneous angle between time steps using MATLAB.

### In situ bright-field videography to measure detailed body kinematics during swimming

To measure details of zooid kinematics, we used an in situ bright-field camera system with a camcorder (Sony AX100) in an underwater housing (Gates Underwater Products) and collimated illumination (Bigblue Dive Lights) oriented directly into the camera lens (*39*). Bright-field images provided detailed morphometric information on the movement of individual zooids and their siphons. The angle of the excurrent siphon was defined by measuring the orientation of the siphon relative to the linear axis of the chain from the oral-aboral (lateral) view of the zooids (ImageJ). The siphon angle was defined as the shortest path perpendicular to the center of the siphon opening to the colony axis.

### Swimming wake structure from in situ stereovideography with dye

Capturing jet wakes from a 3D colony is challenging in the laboratory, so we used fluorescein dye as a passive fluid tracer in the field. Fluorescein powder (Sigma-Aldrich) was preloaded into a micropipette tip and at the start of the dive, the micropipette tip was filled with ambient seawater. Dye was applied in 5- to 10-μl increments into the incurrent siphons of swimming colonies. The resultant jet wakes were filmed with the in situ stereo system. Jet wakes were subsequently analyzed to determine the overall shape and jet angle relative to the chain axis.

### Swimming wake speeds from laboratory particle image velocimetry

Despite the challenges associated with capturing high-speed wakes of swimming salp colonies in the laboratory, we were able to collect a small number of laboratory sequences using laboratory PIV [following (*29*)].
