# Supplementary material for: Spinning and corkscrewing of oceanic macroplankton revealed through in situ imaging
Source: Sci Adv. 2024 May 15;10(20):eadm9511. doi: 10.1126/sciadv.adm9511 (PMC11095445; doi:10.1126/sciadv.adm9511)
Supplement: Supplementary file 1 — Fig. S1 Legends for movies S1 to S6 Legend for data S1 [file sciadv.adm9511_sm.pdf]

Science Advances
AAAS

Supplementary Materials for

Spinning and corkscrewing of oceanic macroplankton revealed through in situ imaging

Kelly R. Sutherland *et al.*

Corresponding author: Kelly R. Sutherland, ksuth@uoregon.edu

Sci. Adv. **10**, eadm9511 (2024)
DOI: 10.1126/sciadv.adm9511

The PDF file includes:

Fig. S1
Legends for movies S1 to S6
Legend for data S1

Other Supplementary Material for this manuscript includes the following:

Movies S1 to S6
Data S1

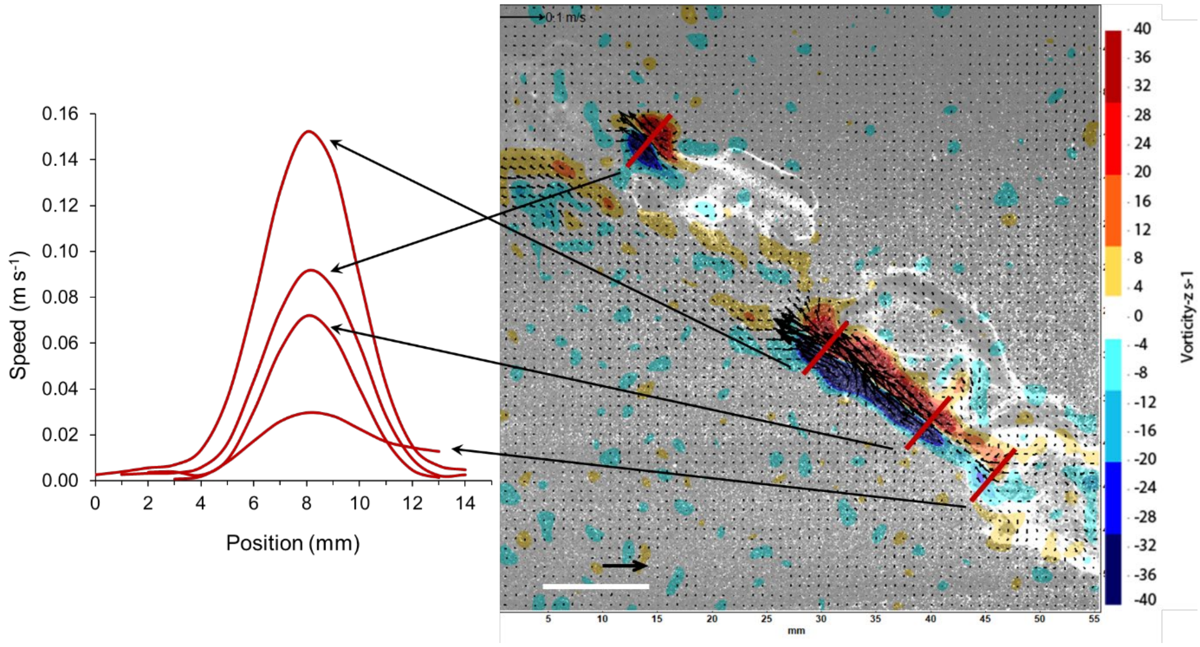

Fig. S1.

Transects through *I. cylindrica* jet wakes showing speed at different points along fully developed jet wake (lower right) and a developing jet wake (upper left). Jet speed reaches a maximum of 0.15 m s^{-1} in the fully developed wake. Scale bar is 1 cm. Vector scale is 10 cm s-1.

Movie S1.

Animation of *I. cylindrica* chain reconstructed from laser scanning showing three-dimensional architecture. The animation plays through twice. The first playthrough is unannotated and the second playthrough shows the dorsoventral zooid angles in yellow and a lateral siphon angle in lavender.

Movie S2.

In situ darkfield videography showing a shorter salp chain spinning around a linear axis and a longer chain swimming in a helix. The spinning dancer illusion makes the spinning direction challenging to discern. As perceived from the posterior end, the first chain is spinning clockwise.

Movie S3.

Animations reconstructed from in situ stereovideography showing spinning and helical swimming. The stereovideography makes it possible to determine direction of spinning.

Movie S4.

In situ brightfield videography played 16.7x slower shows time-varying pulsing by individual zooids in a chain. Excurrent siphons form their lowest angles relative to the chain axis during jetting. Figure 4 indicates how angles were measured relative to the chain axis.

Movie S5.

In situ fluorescein dye visualization shows that jets emerge at a low angle and fluid is rolled up into vortex rings. Jets between neighboring zooids are non-interacting.

Movie S6.

Lab particle image velocimetry (PIV) played 5x slower shows time varying jets reaching maximum speeds of 15 cm s^{-1}. The first playthrough shows vorticity contours and velocity vectors. The second playthrough shows speed contours.

Data S1. (separate file)

All *I. cylindrica* colonies imaged and parameters used to describe morphology, kinematics and fluid mechanics of swimming. Table includes original filenames, geographical location, and imaging method for each data entry.
